# The Aetiological Role of Human Papillomavirus in Oesophageal Squamous Cell Carcinoma: A Meta-Analysis

**DOI:** 10.1371/journal.pone.0069238

**Published:** 2013-07-24

**Authors:** Surabhi S. Liyanage, Bayzidur Rahman, Iman Ridda, Anthony T. Newall, Sepehr N. Tabrizi, Suzanne M. Garland, Eva Segelov, Holly Seale, Philip J. Crowe, Aye Moa, C. Raina MacIntyre

**Affiliations:** 1 School of Public Health and Community Medicine, UNSW Medicine, University of New South Wales, Sydney, Australia; 2 Department of Microbiology and Infectious Diseases, The Royal Women’s Hospital, Melbourne, Australia; 3 Department of Medicine, St. Vincent’s Hospital Clinical School, University of New South Wales, Sydney, Australia; 4 Department of Surgery, Prince of Wales Hospital Clinical School, University of New South Wales, Sydney, Australia; Kagoshima University Graduate School of Medical and Dental Sciences, Japan

## Abstract

**Background:**

The aetiological role of human papillomavirus (HPV) in oesophageal squamous cell carcinoma (OSCC) has been widely researched for more than three decades, with conflicting findings. In the absence of a large, adequately powered single case-control study, a meta-analysis of all available case-control studies is the most rigorous way of identifying any potential association between HPV and OSCC. We present the first global meta-analysis of case-control studies investigating the role of HPV in OSCC.

**Methods:**

Case-control studies investigating OSCC tissue for presence of HPV DNA were identified. 21 case-control studies analyzing a total of 1223 cases and 1415 controls, met our inclusion criteria. HPV detection rates were tabulated for each study and all studies were assessed for quality. The random effects method was used to pool the odds ratios (OR).

**Results:**

From all OSCC specimens included in this meta-analysis, 35% (426/1223) were positive for HPV DNA. The pooled OR for an HPV-OSCC association was 3.04 (95% CI 2.20 to 4.20). Meta-regression analysis did not find a significant association between OR and any of the quality domains. Influence analysis was non-significant for the effect of individual studies on the pooled estimate. Studies conducted in countries with low to medium OSCC incidence showed a stronger relationship (OR 4.65, 95% CI 2.47 to 8.76) than regions of high OSCC incidence (OR 2.65, 95% CI 1.80 to 3.91).

**Conclusions:**

Uncertainty around the aetiological role of HPV in OSCC is due largely to the small number and scale of appropriately designed studies. Our meta-analysis of these studies suggests that HPV increases the risk of OSCC three-fold. This study provides the strongest evidence to date of an HPV-OSCC association. The importance of these findings is that prophylactic vaccination could be of public health benefit in prevention of OSCC in countries with high OSCC incidence.

## Introduction

Oesophageal cancer is the eighth most common malignancy worldwide, with an annual incidence of over 500,000 [1]. It is responsible for 406,000 deaths per annum, making it the sixth highest cause of cancer-related mortality globally [1]. Of the various histological subtypes of oesophageal cancer, oesophageal squamous cell carcinoma (OSCC) accounts for a majority and is predominant in developing countries [2].

The multifactorial aetiology of OSCC is thought to contribute to its highly variable incidence rates across the globe with up to a 500 fold difference between high risk areas such as the Transkei region of South Africa, the Caspian Littoral of Iran and Northern China and low incidence regions such as Western Africa [1], [3]. Potential risk factors for OSCC have been described previously [3], [4] and besides those which are well established, such as smoking and excessive alcohol consumption, perhaps no other factor is of more interest and relevance than human papillomavirus (HPV).

In 1982, Syrjänen observed characteristic HPV related morphological changes, usually found in condylomas, in both benign and cancerous oesophageal tissue [5], first generating the hypothesis that HPV could potentially be involved in the pathogenesis of oesophageal malignancies. This was supported by subsequent immunohistochemical studies, which demonstrated HPV structural proteins within oesophageal lesions of South African, Japanese and Chinese cohorts [6], [7]. Any potential association of HPV to oesophageal carcinoma appears to be restricted to the OSCC subtype. In the last thirty years, evidence for the role of HPV in OSCC has been sought in animal models, morphological, serological and in vitro studies as well as by assessment for viral presence in oesophageal squamous papillomas and malignant tissue [3]. Testing of human OSCC tissue for the presence of HPV DNA and RNA to suggest transcription and activity of HPV, is the most reliable method of investigation for any potential link [3], [4]. However, despite an increasing volume of research, inconsistent rates of HPV detection in OSCC tissue, ranging from 15–80% have been reported in different studies [3], [4], providing conflicting results. A combination of inter-laboratory variations in testing methods, differences in sensitivity and target of assays, the use of different types of test specimens i.e. tissue biopsies, surgical resection specimens, balloon cytology and serology as well as variations in the methods used for histological classification of oesophageal malignancy and the presence of multiple co-factors associated with the disease process, may contribute to the variable reports of HPV DNA detection [4].

While the role of certain oncogenic HPV types in some oropharyngeal and anogenital cancers has been acknowledged by the International Agency on Research on Cancer (IARC), there has been no consensus about a potential aetiologic relationship between HPV and OSCC [8].

The development of prophylactic HPV vaccines, Gardasil® (Merck Sharpe Dohme) and Cervarix® (GlaxoSmithKline), in recent years has been of significant benefit in the fight against cervical cancer and other anogenital cancers. The association of HPV with non-smoking-related head and neck cancers, especially tonsillar, is also now firmly established and the impact of vaccination on the incidence of these cancers is awaited. Clinical trials of HPV vaccines against non-cervical cancers are lacking. However prophylactic efficacy of the quadrivalent HPV vaccine has proven its efficacy on HPV-related vulvar and vaginal lesions with efficacy from approximately 35%–94% in randomised controlled trials [9]–[11]. Thus, by proof of principle, they may well prevent other HPV-related cancers to varying degrees. This, plus the typically late clinical presentation and poor prognosis of OSCC is a good reason to resolve the question about the role of HPV in OSCC, particularly for countries where OSCC is a leading cause of cancer death.

Case-control is the most suitable methodology for the investigation of an HPV-OSCC association. Yet the majority of studies carried out in this area to date have been small, poorly designed case series unsuitable to answer any questions of aetiology, because measures of association cannot be calculated without a control group for comparison. However, a number of small-scale case-control studies have been conducted. We performed a meta-analysis of case-control studies on this topic.

## Methods

### Literature Search

A literature search of the MEDLINE, PUBMED and EMBASE databases was performed to identify published studies in peer-reviewed journals, which investigated for a potential association between HPV and OSCC. Key search terms were “human papillomavirus”, “papillomavirus infections”, “(o)esophageal neoplasms”, “carcinoma, squamous cell”. Only English language papers were included. Articles were sourced from the earliest dates available in each database until February 2012. In addition, reference lists from all case-control studies were reviewed and hand searches of key journals publishing in this area (Annals of Oncology, Lancet Oncology, Anticancer Research, Gastroenterology, International Journal of Cancer, BMC Cancer, Diseases of the Esophagus, Cancer Epidemiology Biomarkers & Prevention and Journal of Clinical Pathology) were performed to retrieve any articles, which were not electronically indexed. No additional data was identified in searches for unpublished papers and abstracts on this topic.

### Selection Criteria

All published studies identified were reviewed by one author (SSL) and included if they examined OSCC and normal oesophageal tissue for the presence of HPV DNA. Animal models, reports on morphology, in vitro and serological studies were excluded. A total of 1223 OSCC and 1415 oesophageal control specimens were tested for the presence of HPV in the 21 case-control studies included in this meta-analysis.

Cases were defined as patients with a histological diagnosis of OSCC and controls were described as healthy subjects with no pre-existing or concurrent chronic medical conditions. There is a potential for cross-contamination and spread of HPV from tumour tissue to adjacent non-malignant oesophageal tissue, creating false positive results in detection of HPV DNA in non-tumour tissue. As a result, 19 articles that classified control tissue as macroscopically normal oesophageal tissue adjacent to the OSCC tumour did not qualify for this meta-analysis.

### Data Extraction

Summative information from the 21 case-control studies was recorded (Table 1) as follows:

**Table 1:** Case-control studies examining HPV DNA in OSCC^a^.

REFERENCE	COUNTRY	HPV DETECTON METHOD	HPV TYPES DETECTED	POSITIVE NO OF CASES (%)	POSITIVE NO OF CONTROLS (%)	OR (95% CONFIDENCE INTERVAL)^1,2^	P VALUE	TOTAL QUALITY SCORE^3^
Williamson *et al.*,1991 [43]	S. Africa	PCR	Various	6/14 (43)	6/41 (15)	4.38 (1.11–17.18)	0.0272	46
Cooper *et al*., 1995 [30]	S. Africa	ISH	6,11,18,31,33	25/48 (52)	0/2 (0)	Incalculable	0.1489	52
Khurshid *et al.*, 1998 [34]	Japan	PCR	CP,16,18	17/27 (63)	3/12 (25)	5.1 (1.11–23.37)	0.0286	60
Agarwal *et al.,* 1998 [26]	India	ISH, IHC	16,18	19/30 (63)	2/10 (20)	6.91 (1.24–38.52)	0.0175	33
Farhadi *et al*., 2005 [31]	Iran	PCR	16,18	8/38 (21)	5/38 (13)	1.76 (0.52–5.97)	0.3608	44
Antonsson *et al.,* 2010 [27]	Australia	PCR	16, 35	8/222 (4)	0/55 (0)	Incalculable	0.1531	61
Fidalgo *et al*., 1995 [32]	Portugal	PCR	16,18	9/16 (56)	0/10 (0)	Incalculable	0.0034	39
Lenhart *et al.,* 1991 [37]	USA	ISH, IHC	6,11,16,18,31,33	4/12 (33)	0/12 (0)	Incalculable	0.0285	54
Koh *et al*., 2008 [35]	Korea	PCR	16	0/102 (0)	0/40 (0)	Incalculable	Incalculable	35
Lambot *et al*., 2000 [36]	Belgium	PCR	CP	1/21 (2)	0/5 (0)	Incalculable	0.6188	22
Benamouzig *et al*., 1992 [29]	France	ISH	6,11,16,18,31,33	4/12 (33)	1/24 (4)	11.5 (1.11–118.71)	0.0171	35
Ashworth., 1993 [28]	UK	ISH	6,11,16,18,31,33	0/4 (0)	0/10 (0)	Incalculable	Incalculable	28
Weston *et al*, 2003 [42]	Brazil	HCII	HR & LR	1/40 (2.5)	1/10 (10)	0.23 (0.01–4.05)	0.2790	44
Souto Damin *et al*., 2006 [41]	Brazil	PCR	16,18	26/165 (16)	0/26 (0)	Incalculable	0.0294	47
Lyronis *et al*., 2005 [40]	Greece	PCR	16,18,other	17/30 (56)	6/27 (22)	4.58 (1.44–14.59)	0.0081	44
Li *et al.*, 2001 [38]	China	PCR, ISH	16	2/2 (100)	66/112 (59)	Incalculable	0.2406	53
Guimaraes *et al*., 2001 [33]	China	PCR	CP	2/32 (6)	4/57 (7)	0.88 (0.15–5.11)	0.8898	65
Cao *et al.*, 2005 [20]	China	PCR	16,18	207/265 (78)	203/357 (57)	2.71 (1.89–3.88)	<0.0001	46
Gao *et al.,* 2006 [21]	China	ISH	nil	0/4 (0)	61/475 (13)	Incalculable	Incalculable	67
Liu *et al.,* 2010 [39]	China	PCR	16	35/69 (51)	2/32 (6)	15.44 (3.42–69.70)	<0.0001	46
Zhang *et al.,* 2010 [44]	China	PCR	16,18,58	35/70 (50)	20/60 (33)	2.00 (0.98–4.08)	0.0552	55

OR – odds ratio; CP – consensus primers; HCII – Hybrid Capture 2; IHC – immunohistochemistry; ISH – in situ hybridization; LR – Low–risk HPV types; HR- High risk HPV types; PCR – Polymerase chain reaction. ^a^Some of the data included in the table have been described in one of our previous publications [4].^1^All odds ratios in this table are unadjusted and have been calculated using STATA, except for 2 studies (Cao et al and Gao et al) where authors of the respective paper calculated adjusted odds ratios.^2^Some papers have ORs and p-values deemed as ‘incalculable’ due to one or more of the four components for OR calculation being a zero value. ^3^Maximum possible total score for a study was 100.

- *General information* – Name of first author, year of publication, country from which cases and controls were sourced;
- *Study design* – Case-control methodology, HPV detection method(s) utilised, number of cases and controls studied;
- *Exposure assessment* – Types of HPV detected;
- *Findings* – Number of HPV positive cases and controls detected;
- *Analysis* – Methodology, if any, of adjustment for confounding factors, calculation of odds ratios (OR) with 95% confidence intervals (CI) and p-values.

The study did not involve collection of raw data from individual researchers, as the data required to conduct the meta-analysis were present in the published papers.

### Quality Assessment

Existing tools for quality assessment of meta-analysis were not used in this study as they do not adequately address specific quality aspects considered important in the investigation of an HPV-OSCC association. Quality assessment was performed using a standardised scoring instrument developed by the authors (BR, SL, AN and ST), based on a previously developed quality assessment tool used in a published meta-analysis [12]. The quality assessment tool used in this study was modified to include criteria specific for case-control methodology, taking into account clinical and epidemiological knowledge of HPV infection and oesophageal cancer, as well as guidelines for quality assessment of observational studies [13]–[16]. Details are listed in “Quality scoring form S1”.

Each study was assessed according to our scoring instrument and assigned a score from a maximum of 100 points. The assessment form was comprised of four main sections of quality assessment including selection of study population (40 points), measurement of exposure (HPV) and outcome (OSCC) factors (40 points), adjustment for confounders (15 points) and analysis of results (5 points). Evaluation of selection and measurement issues were given the highest weighting as these are of relatively greater significance in observational studies. The analysis category was allocated the lowest weighting on the basis that a study which is awarded high scores in the three remaining domains of quality assessment could theoretically have data re-analysed. Each of the four main sections were further sub-divided into individually scored items, which were weighted according to relevance for quality assessment and summed to provide the overall section score. For instance, within the measurement category approximately 65% of marks were ascribed to accuracy of exposure (HPV) assessment while the remaining 35% were allocated to measurement of outcome (OSCC) because the specific HPV identification techniques which are utilised are of paramount importance in any study aiming to report the presence of the virus in oesophageal tissue.

The quality evaluation forms were accompanied by a detailed introduction to the various components of assessment and written instructions on how to complete the scoring instrument. Each study was scored in a blinded fashion by two independent assessors (SL and IR). Upon completion of the scoring process, inconsistencies were discussed by the scorers and a single best answer agreed upon. In the instance that an agreement could not be reached, a third scorer (BR) was consulted to resolve the discrepancy. An assessment of inter-observer correlation was conducted.

### Statistical Analysis

Average quality scores and inter-observer agreement on scoring for all studies was assessed by calculating intra-class correlation coefficient, using the SPSS software [17]. All other statistical analysis was carried out using Stata Version 10.1 SE [18]. Odds ratios for all 21 studies were pooled using the random effects model. Between-study heterogeneity was assessed using two statistical parameters: the I^2^ and Q tests. The Q value is a test statistic for heterogeneity of the OR and the I^2^ value describes the percentage of variability across the studies that is attributed to heterogeneity rather than chance [19].

As only two studies [20], [21] reported an OR, we calculated effect estimates for each study using the information on cases and controls provided in the papers. Of the 21 case-control studies, 10 had ORs which were incalculable due to zero values for one or more of the four cell values (a, b, c, d). As per standard practice for meta-analysis in such instances, a value of ‘0.5’ was added to all the cells in the 2×2 table in order to calculate an OR for these studies [22], [23]. Separate analysis was carried out of studies with calculable and incalculable ORs to investigate whether there is a difference between these 2 groups of studies where the HPV-OSCC association is concerned.

Meta-regression analysis of the association between the OR and the total and four quality scoring domains was carried out to investigate the potential effect of study quality on effect estimates. A cumulative meta-analysis was also conducted to investigate the cumulative evidence at the time that each study was published and to show the trend of results over time. The impact of each individual study on the pooled OR was investigated by performing an influence analysis, which omitted one study at a time in the calculation of the summary outcome. Existing publication bias was assessed with Begg’s and Egger’s tests and by examining for irregularities in funnel plots demonstrating the relationship between the individual log ORs and their standard errors [24], [25].

Based on GLOBOCAN guidelines [1] and current literature, studies were grouped into two separate categories by area of OSCC incidence i.e. studies carried out in high OSCC incidence countries and studies carried out in countries with low-medium incidence OSCC. The high incidence countries were China, South Africa and Iran, which are the three countries with the highest OSCC incidence globally. All remaining countries were categorized as low to medium incidence. We undertook a sub-group analysis by region of OSCC incidence to determine whether there is any correlation between the strength of HPV-OSCC association and the incidence rate of OSCC in the study population according to this categorisation (high incidence versus low to medium incidence).

## Results

**Figure 1:**
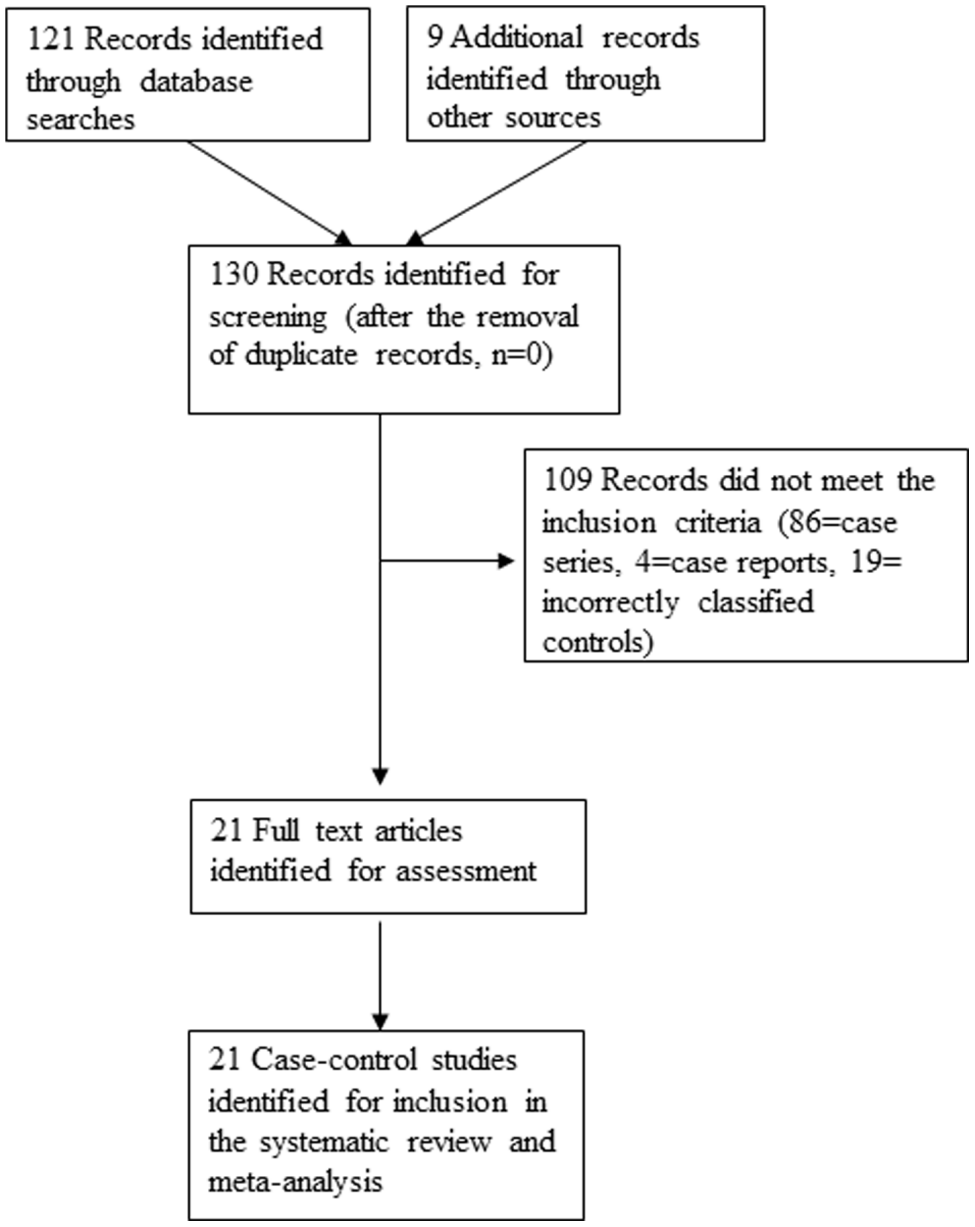
Consort diagram for the study selection.

From literature searches, a total of 130 published studies evaluating a possible HPV-OSCC link were identified. Of these, 86 studies were case series, 4 were case reports and 19 studies examined were ineligible as they classified as controls, specimens such as para-oesophageal tissue, oesophageal tissue from patients with oesophagitis and known head and neck malignancies. A consort diagram outlining the selection of studies for inclusion is shown in Figure 1. Twenty one case-control studies met our inclusion criteria for the meta-analysis [20], [21], [26]–[44] and are described in Table 1. In these studies, a total of 1223 OSCC and 1415 oesophageal control specimens were tested for the presence of HPV. HPV was detected in 35% of cancer samples and in 27% of the control samples.

### Quality Scoring

The averaged quality scores for all case-control studies are summarised by section in Table 2. The main section percentages and where relevant, sub-section percentages referred to below, represent the average percentage of the maximum possible score for that category of quality assessment. The total average percentage for quality assessment for all 21 case-control studies was 46%. Within the Selection category, while the sub-section of ‘study base’ was reasonably well defined in most studies (55%), the relatively poor selection of cases (18%) and controls (14%) resulted in an overall Selection category percentage of only 29% (Table 2). With an average score of 82%, the Measurement component demonstrated the highest level of quality in case-control studies, compared to all other sections. Measurement of outcome (88%) and exposure (78%) were both conducted comparatively well. Adjustment for confounding factors (11%) and statistical analysis of results (10%) both scored poorly. Only five studies [20], [21], [31], [32], [40] adjusted for confounders and odds ratios were calculated in only two studies [20], [21], contributing to the low scores allocated to these two areas of quality assessment. In addition, only 13 studies reported taking measures to ensure quality control or to prevent contamination of samples being tested for HPV [20], [21], [27], [30], [31], [33], [35], [36], [38], [39], [41], [43], [44].

**Table 2:** Average quality scores and inter-observer agreement on scoring for all studies included in the meta-analysis.

	Average (%) of the maximum category quality score (range)	
Categories of quality scoring (maximum points value)	Case-control studies (n = 21)	Inter-cluster correlation (ICCs) for inter-observer agreement (95% CI)
Selection (40 points)	29 (0–67)	0.93 (0.82 to 0.97)
Measurement (40 points)	82 (56–100)	0.53 (−0.11 to 0.81)
Adjustment for confounding (15 points)	11 (0–73)	0.91 (0.77 to 0.96)
Analysis (5 points)	10 (0–100)	0.88 (0.70 to 0.95)
Total (100 points)	46 (22–67)	0.87 (0.62 to 0.95)

The total inter-class correlation (ICC) of 0.87 (95% CI 0.62 to 0.95), showing very good inter-observer agreement, indicates reliability of the developed scoring instrument (Table 2). The ICCs for the four quality assessment sections ranged from 0.53 to 0.93. The relatively low ICC score (0.53, CI −0.11 to 0.81) ascribed to the Measurement section was attributed to the indistinct reporting of specimen storage and retrieval methodology in several studies.

### Pooled Odds Ratios and Meta-analysis

**Figure 2:**
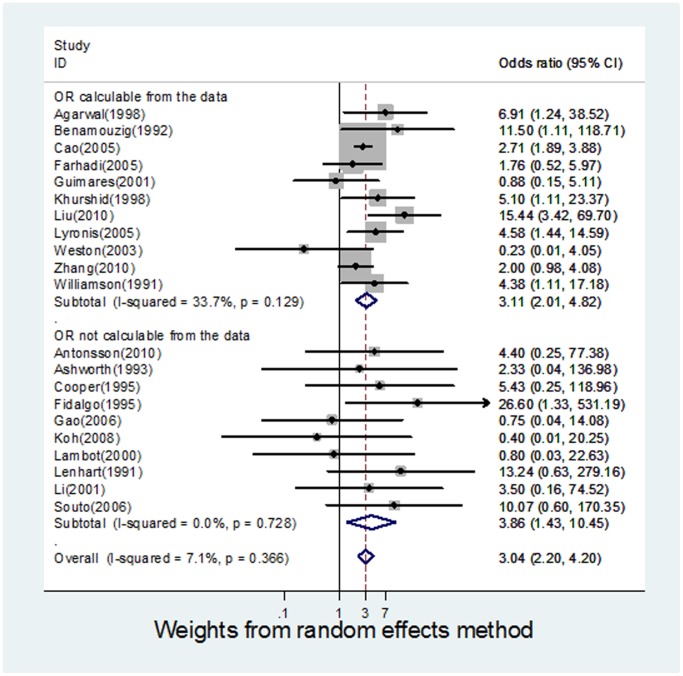
Forest plot for meta-analysis of the association of HPV with oesophageal squamous cell carcinoma in 21 case-control studies.

Individual and pooled OR estimates derived from a random effect model analysis have been illustrated in a Forest plot (Figure 2).

The ORs for the case-control studies analysed, ranged from 0.23 to 26.6. The pooled estimate was 3.04 (95% CI 2.20 to 4.20), indicating a significant association between HPV and OSCC. Cochrane’s Q test for heterogeneity was not significant across all studies (Q = 21.54, p-value 0.366) and the I^2^ value was 7.15%. This can be interpreted as 7.15% of the variation across the studies being attributed to heterogeneity rather than chance. An I^2^ value of 25% is generally considered to be low, suggesting that it is less likely that any variability in this meta-analysis is due to chance [19].

### Meta-analysis of Studies based on Calculable and Incalculable OR

There were 11 studies [20], [26], [29], [31], [33], [34], [39], [40], [42]–[44] for which ORs could be calculated using data provided in the papers. A meta-analysis of these studies alone (Figure 2) produced a pooled OR of 3.11 (95% CI 2.01 to 4.82) and an I^2^ value of 33.77. The remaining 10 studies [21], [27], [28], [30], [32], [35]–[38], [41] required manipulation of data using standardised techniques, in order to calculate individual ORs, were also analysed as a separate group (Figure 2), resulting in a pooled OR of 3.86 (95% CI 1.43 to 10.45), and an I^2^ value of 0. Therefore, independent analysis of both sets of studies showed a significant HPV-OSCC association, independent of the presence of the other subset, adding further weight to evidence for an HPV-OSCC association.

### Meta-regression

The meta-regression analysis investigated the association between study-specific ORs and the quality scoring domains i.e. Selection, Measurement, Confounding, Analysis and overall study quality, to test whether any of the quality assessment sections were associated to the ORs from the individual studies (Table 3). The regression coefficients for all the quality assessment sections were non-significant, with no evidence that the ORs are influenced by any of the quality domains or the overall study quality.

**Table 3:** Results of meta-regression analysis of OR for HPV-OSCC association on scores from quality domains of all studies included in the meta-analysis.

Item in quality score	Regression co-efficient (95% CI)	P-value
Selection	−0.0165737 (−0.05 to 0.02)	0.325
Measurement	0.0433383 (−0.03 to 0.12)	0.274
Confounding	−0.028375 (−0.11 to 0.06)	0.521
Analysis	−0.0407042 (−0.15 to 0.06)	0.449
Total score	−0.0261726 (−0.07 to 0.01)	0.212

### Cumulative Meta-analysis

**Figure 3:**
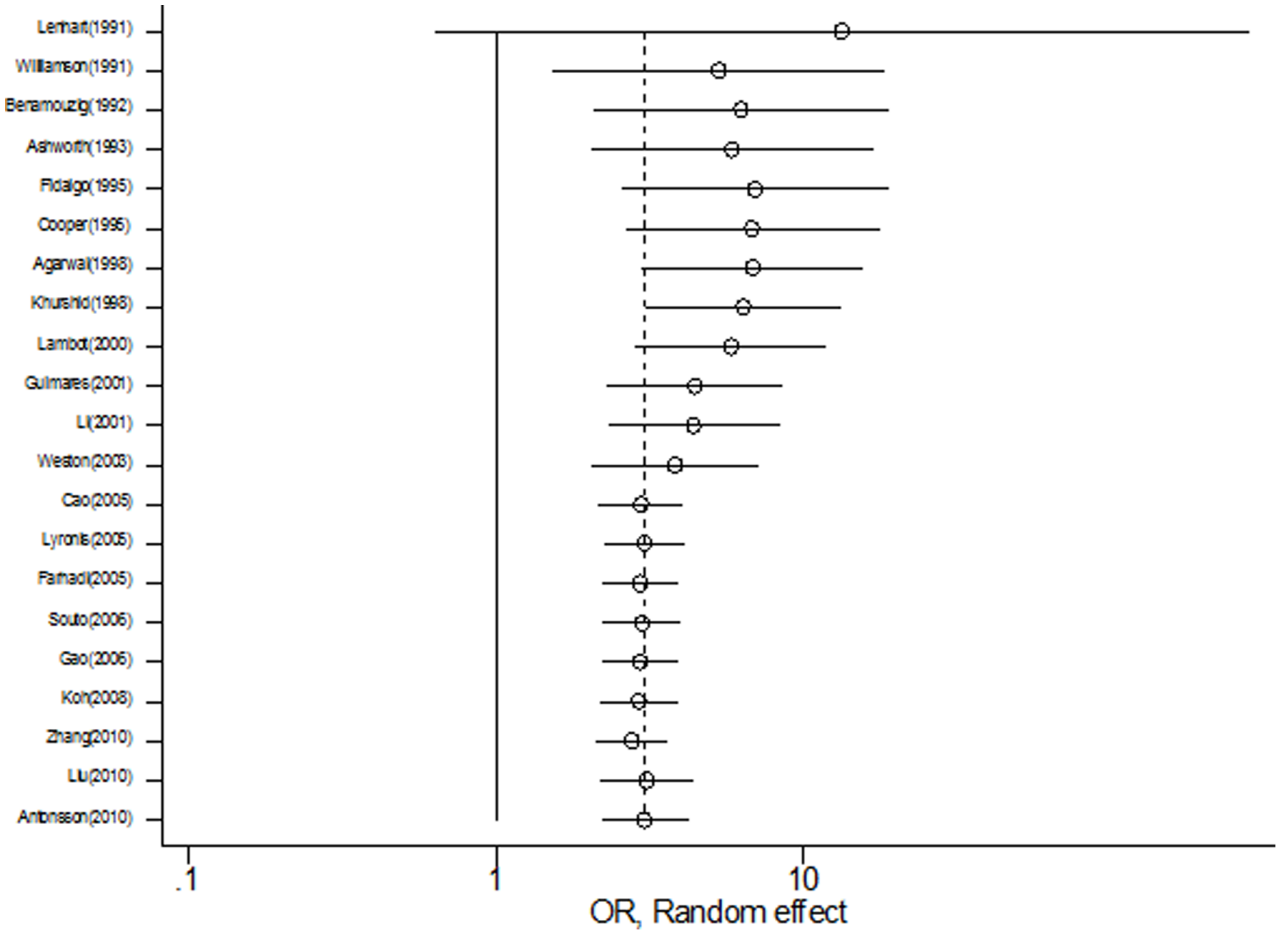
Cumulative meta-analysis of case control studies for the evidence of HPV involvement in OSCC.

A cumulative random-effects meta-analysis of the 21 studies revealed the trend of results over time. All studies demonstrated a positive HPV-OSCC association. Earlier studies indicated a stronger association of HPV with OSCC, with the earliest study in 1991 [37] showing a cumulative estimate of 13.24 (95% CI 0.63 to 279.16), compared to the most recent study in 2010, which had a cumulative estimate of 3.04 (95% CI 2.20 to 4.20). While the demonstrated HPV-OSCC link is still positive in studies from 2005 and onwards, cumulative studies have decreased the confidence interval for the summary estimate (Figure 3).

### Influence Analysis of Individual Studies

**Figure 4:**
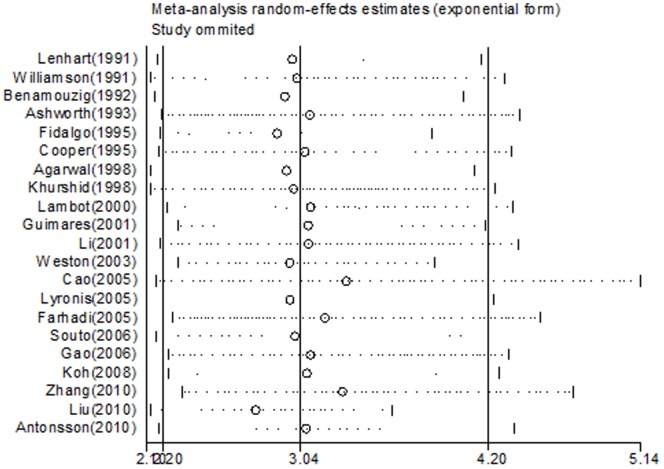
Influence analysis for individual studies on the summary effect.

The meta-analysis result of the pooled OR was not significantly affected by omission of any of the 21 individual studies analysed (Figure 4).

### Publication Bias

**Figure 5:**
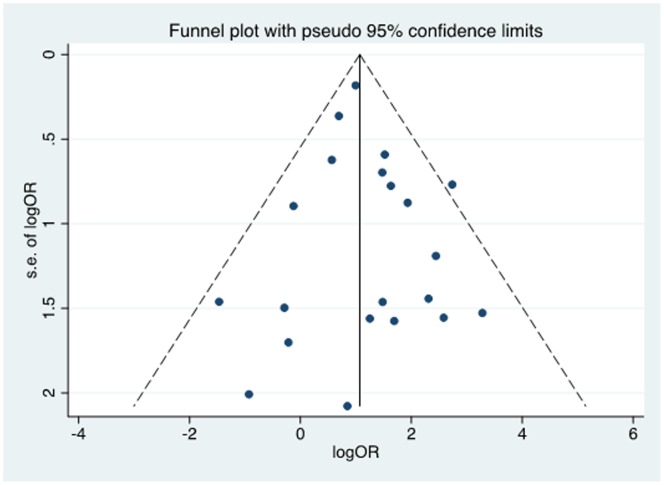
Funnel plot for analysis results of publication bias.

There was no evidence of publication bias as demonstrated by the non-significant p-values for both Begg’s (0.39) and Egger’s test (0.48), and near-symmetric funnel plot (Figure 5).

### Sub-group Analysis by Region of OSCC Incidence

**Figure 6:**
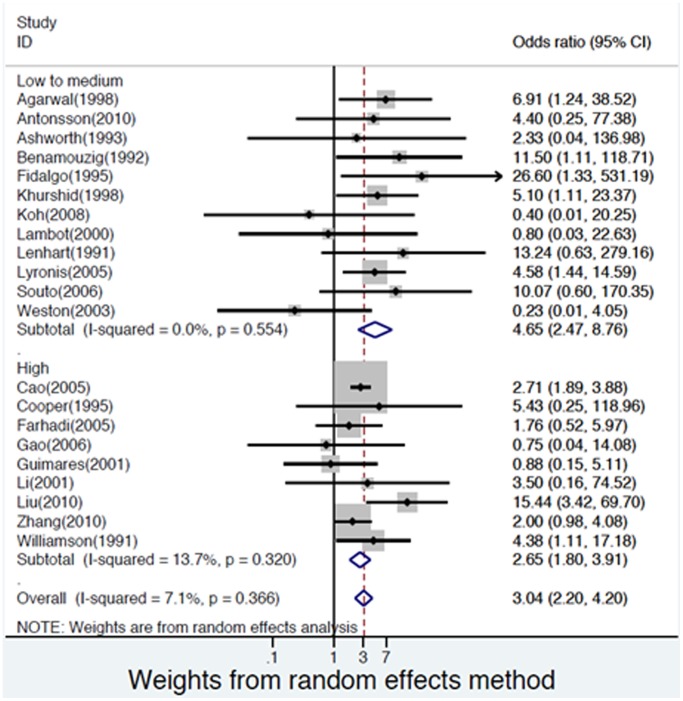
Forest plot and meta-analysis by region of OSCC risk level.

Nine studies analysed OSCC specimens from high incidence OSCC regions [20], [21], [30], [31], [33], [38], [39], [43], [44], while the remaining 12 studies recruited OSCC patients from low to medium risk OSCC populations [26]–[29], [32], [34]–[37], [40]–[42]. The HPV-OSCC association was positive and significant in both high (pooled OR 2.65, 95% CI 1.80 to 3.91) and low to medium (pooled OR 4.65, 95% CI 2.47 to 8.76) incidence regions as demonstrated in Figure 6. The HPV-OSCC association was found to be relatively stronger in low to medium incidence OSCC countries compared to high OSCC countries.

## Discussion

This meta-analysis demonstrates that HPV increases the risk of OSCC by three-fold and provides the strongest evidence to date of a potential role for HPV in the aetiology of OSCC. While causation cannot be clearly established, it is a major step toward resolving a longstanding uncertainty. With most cancers, the question of aetiology can only be addressed by an observational epidemiologic study, since it is not ethical or feasible to use any other study design to determine aetiology. To date, the association of smoking and lung cancer is a statistical association found in cohort and case control studies, such as the pivotal case-control and cohort studies of Doll and Hill, which to this date remain the strongest evidence linking smoking to lung cancer [45], [46]. This analysis has excluded chance as an explanation for the observed association, which supports causality. We have pooled and analysed the data from all available case-control studies, which have been small scale and unable to make a definitive contribution as single studies. The confusion, delay and continued controversy of the role of HPV is due to inappropriate evaluation of the evidence. The availability of an effective HPV vaccine and the potential therein to prevent HPV-associated cancers [9]–[11], provides a public health impetus to resolve this question, particularly for countries of high OSCC incidence.

Despite there being 130 studies on the topic, the question of whether HPV plays a role in the aetiology of OSCC has been poorly studied. Suboptimal study design, heterogeneous and small-scale studies, inconsistent laboratory methods for HPV detection and variations in specimen retrieval and storage have all contributed to the lack of clarity and inability to resolve this question to date. We found that the majority of the 130 studies identified are case series, which are unable to answer questions of aetiology because a measure of association cannot be calculated without a control group [13].

In conducting this meta-analysis, we discovered that there are very few case-control studies done on the subject, and of the 21 studies most are small scale, with the largest recruiting only 265 subjects. Case-control study design is particularly time-efficient and economical when investigating diseases with long latency periods such as OSCC, since the case subjects have already been diagnosed with the condition at the start of the investigation [13]. Case-control methodology also permits simultaneous and independent investigation of multiple aetiologic components, which is advantageous in the assessment of diseases such as OSCC which have several risk factors [47]. Only 16% (21/130) of studies investigating the role of HPV in OSCC were case-controls.

Interestingly, of the 21 case-control studies, all presented data from which a measure of association such as an OR could be calculated, but only 2/21 papers presented an OR calculated by the authors. This again highlights the lack of multi-disciplinary, methodological expertise which has gone into evaluating this research question. Our study is therefore a significant contribution to advancing knowledge by utilising the available case control studies to show a convincing and strong statistical association of HPV with OSCC. HPV was detected in a significantly higher proportion of OSCC samples than control tissue, and if this result is representative of the general population, it may be possible that HPV acts as a carcinogen or important co-factor in the pathogenesis of a significant proportion of OSCC cases across the globe.

We believe this is a major step forward in the understanding of this subject, supporting an aetiological role of HPV in OSCC, and that there is some ethical imperative to resolving the question, given the availability of a preventive HPV vaccine, which could have a major impact in preventing OSCC in high incidence countries such as South Africa, Iran and China [16] where OSCC is a major contributor to cancer deaths.

The significant pooled OR (3.042, 95% CI 2.2–4.2) which we obtained from the random effects analysis, supported by an I^2^ value of 7.1%, is strongly indicative of an aetiological role for HPV in OSCC. This result is further validated by our analysis of all subcategories. Our influence calculations demonstrate that no single study on its own, affected the summary effect significantly more or less than any other study included in the meta-analysis, which further supports the robustness of the study. In addition, there was no evidence of publication bias and our meta-regression analysis revealed that study quality did not influence the ORs. The required manipulation of data for studies with incalculable ORs, also did not affect the effect measure, as separate analysis of both studies with calculable and incalculable ORs yielded independently significant pooled ORs suggestive of an association between HPV and OSCC. All these results in combination, support and strengthen our finding of an HPV-OSCC link.

General trends have shown that subjects from areas with a relatively higher incidence of OSCC demonstrate higher rates of HPV DNA detection in OSCC tissue, in comparison to those from regions of low OSCC incidence [3]. Interestingly, our sub-group analysis by incidence of OSCC region did not support this finding. Although our results indicate a positive HPV-OSCC association in both high and low-medium incidence areas for OSCC, the evidence for a role of HPV in the aetiology of OSCC was relatively stronger in low-medium incidence countries such as Greece, USA and Australia, compared to reports from high incidence regions such as China, Iran and South Africa. Previous reports of higher rates of HPV detection in OSCC tissue from high incidence areas has been based on collective results from 130 studies of differing methodology from case-control and cross-sectional studies to case series and case reports, while our analysis is based solely on data from case-control studies. Regions with relatively lower rates of OSCC incidence are primarily high-income countries, possibly correlating with increased resources, more advanced laboratory techniques and methodology for investigations. These factors may explain our findings of higher rates of HPV detection in OSCC tissue from areas of low-medium OSCC incidence, compared to high OSCC incidence regions, which are predominantly developing nations.

Our results suggest that while all studies showed a positive correlation between HPV and OSCC, the association was stronger in earlier studies compared to later studies. There is no evidence that this may be due to HPV detection methodology i.e. amplification versus non-amplification of DNA, as studies carried out in the early 1990s also used DNA amplification techniques [32], [43].

A limitation of this study is that most of the available case control studies, in addition to not calculating a measure of association, did not measure potential confounders or effect modifiers or adjust for their effects. Age, gender, smoking, alcohol consumption, family history of oesophageal cancer, pre-existing immunosuppression prior to cancer diagnosis, a history of thoracic irradiation, socio-economic status, diets high in red and processed meat, consumption of hot food and beverages, pickled foods and diets low in fresh fruit and vegetables are all confounding for effect-modifying actors which may have an impact on the analysis of a HPV-OSCC link. The study did not involve collection of individual raw data, however, all provided aggregate data categorised into exposed and ill; exposed and not ill; non-exposed and ill; and non-exposed and not ill; therefore allowing the meta-analysis to be done. Only five of the studies presented (or purported to collect) data on co-factors, effect modifiers or confounders such as smoking or alcohol, so adjustment for these factors was not possible in the meta-analysis, even if raw data were used. We are not aware of any known confounders which could result in such a strong association of HPV with OSCC. Any future single case control study to address this issue would need to be substantially larger than any of the currently published studies in order to add to the knowledge in light of this meta-analysis. We would recommend that such a study should collect data on potential confounders and effect modifiers and that these be adjusted for when examining the effect of HPV. A final limitation is the varying ratio of cases to controls in the studies, which suggest ad-hoc or convenience-based design rather than carefully planned study designs. However, we included all 21 studies meeting the inclusion criteria, regardless of the ratio of cases to controls.

Based on the findings outlined in GLOBOCAN 2008 and the cancer registry data from Cancer Incidence in Five Continents, it has been estimated that HPV is responsible for approximately 5.1% of the global cancer burden and contributes 20%–50% of non-anogenital cancers [1], [2], [48] and is thought to be the primary oncogenic factor in more than 70% of cervical cancer cases [49]. In countries such as China, however, oesophageal malignancy is the fourth leading cause of cancer-related deaths and 99% of all oesophageal cancers in China are of the squamous cell carcinoma subtype [50]–[52]. Of the two prophylactic HPV vaccines which are currently available, Cervarix® is a bivalent vaccine, which targets the most common oncogenic HPV types 16 and 18 [49]. Gardasil® is a quadrivalent vaccine, which immunises against both types 16 and 18 as well as types 6 and 11 (commonly associated with benign conditions such as genital warts). With the exclusion of Australia, where adolescent girls have received the quadrivalent vaccine since 2007, and which will be the first country to implement vaccination for boys in a school based government funded program [53], at present most countries with prophylactic HPV vaccination programs target only young girls prior to sexual debut. Most low and middle income countries, however, do not have publicly funded HPV vaccination programs. Growing evidence for the association of HPV with a number of other anogenital and oropharyngeal cancers supports proposals to extend coverage to include vaccination of males as well. A cost-effectiveness analysis of the population benefit of HPV vaccination is needed to address the potential additional impact of preventing non-cervical malignancies in boys and girls, particularly in high-incidence OSCC countries such as China, Iran and South Africa.

Ours is the first meta-analysis of case-control studies on this topic. A recent meta-analysis of HPV and OSCC did not restrict analysis to any particular study design [54] and concluded that the inconsistency in HPV detection rates between studies can be attributed to the geographic region of origin of study population, rather than HPV detection method. The authors stipulated that HPV is only a significant aetiological factor in OSCC incidence in high-risk regions. These findings are in contrast to our results, which suggest that HPV is of greater significance in the aetiology of OSCC in low to medium risk regions.

### Conclusions

This study is the most definitive contribution to date about a question which has defied answers for 30 years: the association of HPV with OSCC. We found, with a significant, robust and strong statistical measure of association, that HPV is associated with a 3-fold increase in the risk of OSCC. There is an imperative for this study to be considered by IARC because of the availability of a prophylactic vaccine against HPV, the late presentation and poor prognosis of OSCC and a large burden of OSCC mortality in many countries. It further adds to the support of HPV vaccination as a cancer-preventing vaccine for children of both genders, to broaden the preventive targets of the vaccine.

## Supporting Information

Quality scoring form S1 (DOCX)
