# Supplementary material for: The Aetiological Role of Human Papillomavirus in Oesophageal Squamous Cell Carcinoma: A Meta-Analysis
Source: PLoS One. 2013 Jul 24;8(7):e69238. doi: 10.1371/journal.pone.0069238 (PMC3722293; doi:10.1371/journal.pone.0069238)
Supplement: Quality scoring form S1 — (DOCX) [file pone.0069238.s001.docx]

**Quality scoring system for case-control studies of human papillomavirus and oesophageal squamous cell cancer**

The objective of this quality scoring system is to evaluate in a standardized manner the quality of observational epidemiological studies into the association between exposure to oncogenic types of human papillomavirus (HPV) and development of oesophageal squamous cell carcinoma (OSCC). The score assigned to each study summarizes the overall quality of that study, giving the most emphasis to the quality of subject selection and exposure (i.e. HPV) assessment. Sub scores for the quality of specific components of studies are also available if needed for analysis.

A total score of 100 has been allocated, covering four broad areas of case-control study design. Most weight has been given to the Selection and Measurement issues (40 points for each), as these are the key areas of any observational study. Of the remaining 20 points, 15 have been allocated to the issue of Confounding and the remaining five points are allocated to the Analysis. The analysis section has been given the least weight because if the other three sections have good quality but the analysis is not carried out properly, then the data could, in theory, be reanalyzed.

To assign points for an individual characteristic relevant to quality, the assessor makes a judgement about which of the categories best describes the specific study characteristic. If there is a range of 0 to 10 for a characteristic, then 10 points are allocated for excellent quality, five for moderate quality and zero for very poor quality. These cut-off points are used to guide the allocation of marks.

**Description of some specific quality characteristics**

**Section A: Selection**

1. ***Study base****:* The study base defines the population from which cases and controls are sourced e.g. from a single endoscopy clinic, a district hospital, multiple regional hospitals etc., which may or may not adequately represent the community being investigated. A study base should also define the time period during which samples were collected or during which subjects were recruited e.g. if specimens were collected over a 2 year time period. The first consideration for a case-control study is the study base: specifically, an assessment as to whether it is well defined or not. A study base is well defined if the study population can be easily defined in terms of time, person and place. Therefore for a study to have a well-defined study base, it should clearly identify how cases/controls were selected, which geographic region the samples were collected from and over what period of time they were collected. If the study base is not well defined, the investigator cannot be confident, for example, that all relevant cases have been included, and that non-relevant cases have been excluded, that the controls represent the study base from which the cases arise, or of how representative the included cases are of all cases in the study base. A study with a poorly defined study base is susceptible to bias in the measure of association between disease and exposure factor (i.e. HPV) in any direction.
2. ***Selection of cases****:* Selection bias may occur if the recruited cases are not representative of all the cases in the study base. The inclusion of a random sample of all cases, or the use of all cases, should eliminate bias resulting from the selection of cases. In nearly all situations, incident cases should be used rather than prevalent ones since the latter are survivors and may differ in material ways from all incident cases.
3. ***Selection of controls****:* The controls in a case-control study must come from the population that has produced the cases. They must also accurately represent the exposure experience of the whole study base. A random sample of the whole study base is therefore desirable.
4. ***Non-participants****:* Non-participation of some appropriately selected cases and controls is an important source of bias in selection. A high participation rate is always desirable and increases internal validity of the study. There is no set acceptable cut off point for the rate of participation, but in general less than 20% non-participation is considered good. However, even where there is a very high rate of non-participation, if the non-participants were similar in all material respects to those who remained, the validity of the study should not be affected. On the other hand, even if the rate of participation is high, if the subjects who dropped out differ significantly from those who remained, the study findings may be biased. Often there is little information about subjects who did not participate. For the purpose of this study, we have split this quality item into three sub categories: a participation rate of greater than 80%, 60% to 80%, and less than 60%. Each of these subcategories also has three sub divisions: where the non-participants are similar to the participants, not similar to the participants, or there is no information about the non-participant’s characteristics. A participation rate of >80% with sufficient information about the non-participants to indicate that they are similar to those who remained represents the best quality. A participation rate of <60% where non-participants are not similar to the participants represents the poorest quality. If there is no information about the non-participants, an intermediate score is given. A study with 60-80% participation rate where the non-participants are found to be similar to the participants would be given a greater score than a study with >80% participation rate but where the lost 20% is not similar to the population who remained. This concept will be the guide for assigning scores on this characteristic.

**Section B: Measurement**

1. ***Accuracy of exposure (HPV) measurement***: Exposure measurement is an important issue, particularly for case-control studies. It has therefore been given more weight in the scoring and been broken down into specific subsections for individual scoring.

With regards to consistency, types of measurement error include non-differential and differential error:

Non-differential error occurs when the inaccuracies of measurement are the same between comparison groups e.g. cases and controls. For example, with regards to specimen retrieval for a given study, if all cases and controls are balloon cytology samples. Non-differential measurement error in exposure (i.e. HPV) and outcome (i.e. OSCC) will always lead to bias towards finding no effect.

Differential error occurs when the inaccuracies of measurement are different between comparison groups e.g. cases and controls, leading to bias towards or away from no effect. For example, if all controls specimens are balloon cytology samples but all case specimens are diagnostic biopsies or the case specimens are a mixture of diagnostic biopsies and surgical resection specimens while the control samples are a mixture of cell brushings and balloon cytology samples.

Specimen retrieval

Specimen retrieval methods refer to how the test specimen was obtained i.e. diagnostic biopsy, surgical resection, balloon cytology, cell brushings etc. No specimen retrieval method has been classified as superior to other methods. Instead, the focus is on whether results methodology has remained consistent between and within the case and control groups, thereby assessing comparability of results.

Specimen storage/sampling

Specimen storage prior to testing refers to how specimens were treated following retrieval i.e. fresh frozen, formalin-fixed and paraffin embedded etc. This section has been divided into 2 categories: type and consistency. For type, fresh frozen tissue sampling is classified as superior to archival tissue. For consistency, it is important to ensure that all cases and controls have been sampled in a consistent manner e.g. the best case scenario would be if all cases and all controls are fresh frozen.

Testing methodology

Specimen testing methodology refers to the method used to detect HPV DNA in test specimens i.e. polymerase chain reaction (PCR), in situ hybridization (ISH), immunohistochemistry (IHC) etc. This section has been divided into 2 categories: type and consistency. For type, the hierarchy is as follows: PCR>HCII>ISH>IHC. Regarding PCR the hierarchy of primers used is as follows: SPF10 > GP 5/6 (or GP 5+/6+) > MY09/11 (or PGMY09/11), based on sensitivity.  For consistency, it is important to ensure that all cases and controls have been sampled in a consistent manner e.g. the best case scenario would mean that all cases and all controls are analysed by PCR; and for primer type, both cases and controls analysed using MY09/11.

If two different methods have been used to test all case and control samples, and results for both methods are provided in the paper, in the meta-analysis we will use only the results from the superior method and do so for scoring. For example, if PCR and ISH have both been used to test all the case and control samples and results from both tests have been provided, as PCR is a superior method compared to ISH for HPV detection results obtained from this methodology will be used in the meta-analysis and hence this method will be considered for scoring.

However, if two testing methodologies have been used to test all case and controls samples, but a complete set of results has been provided from only one of the testing methods, then this method should be used for scoring regardless of superiority. For example, if PCR and ISH have both been used to test all the case and control samples but results are only provided for ISH, then ISH should be used for scoring, even ‘though PCR is the superior methodology.

When different methods have been used to test a proportion of the total case or control samples e.g. from a total of 100 cases if PCR is used to test only 30 samples and ISH is used to test the remaining 70 samples; or if PCR is used to test 100 cases and 25 controls and ISH is used to test the remaining 75 controls, the mixed methodology across cases and controls means that results will be incomparable and therefore the score should be ‘0’ as outlined in section B.2.3.

1. ***Accuracy of outcome (OSCC) measurement****:* Accurate outcome measurement is important to ensure that the cases are all OSCC tissue and controls are all normal, non-malignant oesophageal tissue. The most reliable information for histopathology of case and control samples is obtained from lab-based histopathology reports. Other sources of information on histopathology may be obtained from hospital admission records, cancer care centres/clinics – however, these may not be entirely reliable.

**Section C: Confounding**

1. ***Confounding****:* There are two methods to adjust for confounding factors: (i) by individually matching the cases and controls (in a matched case-control study) by the confounding factors (e.g., sex, age etc.) and using appropriate statistical method (e.g. conditional logistic regression) to take into account of the matched factor during the analysis; (ii) by adjusting in a multivariable model for an unmatched case-control study. It is important to assess whether the study has considered and adjusted for all the relevant confounders and whether any adjustment made is appropriate or not. With respect to the latter, if a study adjusts for a variable, which is not known a priori to be a confounder, lying in the causal chain between exposure (i.e. HPV) and outcome (i.e. OSCC), the effect measure may be biased because of the adjustment.

Risk factors do not bias the point estimates but affect their precision, while confounders can affect both. Therefore risk factors have been given a lower weighting than known confounding factors.

*Confounding factors* – may also be risk factors (for either cause/effect, or both)

- Age – HPV, OSCC
- Gender - OSCC
- Smoking – HPV, OSCC
- Alcohol – OSCC

*Risk factors* – may have a confounding effect (risk factor for either cause/effect, or both)

- Family history of oesophageal cancer (OSCC)
- Pre-disease immunosuppression – HPV, OSCC
- Socio-economic status – HPV, OSCC
- Previous history of thoracic irradiation - OSCC
- Dietary intake high in red meat, hot food/beverages, pickled food, low in fresh fruits/vegetables - OSCC

It is important to ensure that the correct methodology for adjustment of confounding factors has been used. Conditional logistic regression must be used if matching has been done, and unconditional logistic regression if there has been no matching. For example if cases and controls have been individually matched for gender, conditional logistic regression analysis must be used for adjustment, using gender as a matching factor.

**Section D: Analysis**

1. ***Analysis****:* A good quality analysis should report the appropriate effect measure and its confidence interval and have the capacity to adjust for potential confounders. In order to avoid double adjustment, it is important to ensure that both matching of cases and controls for a particular factor, as well as statistical adjustment for the same factor during analysis, has not been carried out. If there are probable interacting variables, the effect measure should also be calculated within strata of the interacting variable and, ideally, reported with a confidence interval. Where there may also be a moderate to long interval between first exposure (i.e. HPV) and appearance of the outcome (i.e. OSCC) analyses that allow for this interval should also be done and reported.

**Guide to scoring studies**

**Case-control studies**

Date:­­­­­­­­­­­­­­­­­­­­­­­ Reviewer:­­­­­­­­­­­­­­­­­­­­­­

Paper ID: Study ID:

Matched/Unmatched study:

**Please highlight or circle the option(s) selected:**

**Section A: Selection issues**

**A1. Study base** (i.e. description of time and place from which samples were collected)

**(Select ONE option)**

**Score from 0 to 10**

- Well-defined 10
- Moderately well-defined 5
- Poorly defined 0

**(Maximum score is 10)**

**A2. Cases**:

**A2.1. Selection of cases (select ONE option) Score from 5 to 10**

- - If all cases or a random sample of cases are recruited 10
  - Cases not randomly selected 5
  - No information on selection 5

**A2.2. Participation rate for cases Score from 0 to 10**

(Select either A2.2.1 **OR** ONE option from A2.2.2)

**A2.2.1 – Retrospective case collection**

- All cases were collected retrospectively

(i.e. all case samples were obtained from tissue/tumour bank) 10

- Not all cases were selected retrospectively: proceed to A.2.2.2

**A2.2.2 – Prospective case selection**

- **More than 80% of the approached cases participated**
- Non-participants are similar to the participants 10
- Non-participants are not similar to the participants 6
- No information about the non-participants 6
- **Between 80-60% of the approached cases participated**
- Non-participants are similar to the participants 8
- Non-participants are not similar to the participants 4
- No information about the non-participants 4
- **Less than 60% of the approached cases participated**
- Non-participants are similar to the participants 6
- Non-participants are not similar to the participants 2
- No information about the non-participants 2
- No information on participation rate 0

**A2.2.3 - Mixed retrospective and prospective case selection**

- More than 80% of the approached cases participated 6
- Between 80-60% of the approached cases participated 4
- Less than 60% of the approached cases participated 2
- No information on participation rate 0

***Overall score for section A2 = (A2.1 x A2.2) / 10 (Maximum score is 10)***

**A3. Controls**

**A3.1. Selection of controls** **(select ONE option)** **Score from** **5 to 10**

- - Controls are randomly selected 10
  - Controls not randomly selected 5
  - No information on selection 5

**A3.2. Participation rate for controls Score from** **0 to 10**

(Select either A3.2.1 **OR** ONE option from A3.2.2)

**A3.2.1 – Retrospective control collection**

- All controls were collected retrospectively

(i.e. all control samples obtained from tissue/tumour bank) 10

- Not all controls were collected retrospectively: proceed to A.3.2.2

**A3.2.2 – Prospective control collection**

- **More than 80% of controls approached participated**
- Non-participants are similar to the participants 10
- Non-participants are not similar to the participants 6
- No information about the non-participants 6
- **Between 80-60% of controls approached participated**
- Non-participants are similar to the participants 8
- Non-participants are not similar to the participants 4
- No information about the non-participants 4
- **Less than 60% of controls approached participated**
- Non-participants are similar to the participants 6
- Non-participants are not similar to the participants 2
- No information about the non-participants 2

- No information on participation rate 0

**A3.2.3 - Mixed retrospective and prospective control selection**

- More than 80% of the approached controls participated 6
- Between 80-60% of the approached controls participated 4
- Less than 60% of the approached controls participated 2
- No information on participation rate 0

***Overall score for A3 = (A3.1 x A3.2) / 10 (maximum score is 10)***

***Section A score = (A1 + A2 + A3) × 4 / 3* (**Maximum score is 40)***.***

­­­­­­­­­­

**Section B: Measurement issues**

**B1. Accuracy of outcome (OSCC) ascertainment:**

**B1.1. Validity of the sources of the outcome data - cases Score from 0 to 10**

**(Select ONE option)**

- - All cases were confirmed by histopathology reports 10
  - More than 90% were confirmed by histopathology reports 9
  - Between 30 & 90 % were confirmed by histopathology reports 4-7
  - Less than 30 %were confirmed by histopathology reports 3
  - No information about the proportion confirmed by reports 3
  - Histopathology not checked on any of the cases 0

**B1.2. Validity of the sources of the outcome (OSCC)**

**data for controls (Select ONE option) Score from 0 to 10**

- - All controls were confirmed by histopathology reports 10
  - More than 90% were confirmed by histopathology reports 9
  - Between 30 & 90 % were confirmed by histopathology reports 4-7
  - Less than 30 % were confirmed by histopathology reports 3
  - No information about the proportion confirmed by reports 3
  - Histopathology not checked on any of the cases 0

***Overall score for B1=(B1.1+B1.2) × 0.75 (maximum score is 15)***

**B2. Accuracy of exposure (HPV) measurement:**

**B2.1. Consistency of specimen retrieval methodology Score from 0 to 10**

**(Select ONE option)**

- All cases and controls taken in the same way (100% consistent) 10
- Mixed specimen types
- >90% cases and controls were consistent 7
- 30-90% cases and controls were consistent 2-5
- <30% cases and controls were consistent 1
- No consistency in specimen retrieval between cases and/or

Controls 0

- Not specified 0

**B2.2 Specimen storage/sampling (Select ONE option)**

**Types of specimen storage/sampling Scoring from 0 to 12**

**(all cases and controls)**

- Fresh frozen 12
- Archival 10

- Mixed specimen storage forms
- >90% cases and controls consistent 7
- 30-90% cases and controls consistent 2-5
- <30% cases and controls consistent 1
- No consistency in storage forms between cases and/or controls 0
- Not specified (either method or mixed percentage) 0

**B2.3 Testing methodology**

**Detection Sensitivity by Testing Method Score from 0 to 24**

**(Select ONE option)**

- **PCR**
- SPF10 24
- GP 5/6 (or GP 5+/6+) 23
- MY09/11 (or PGMY09/11) 21
- Mixed PCR primers 19
- PCR primer not specified 19
- HCII 17
- Dot Blot (DB) 13
- ISH 12
- IHC 10
- Mixed specimen testing methodology (HCII,ISH,IHC,PCR,DB)
- >90% cases and controls tested in the same way 7
- 30-90% cases and controls tested in the same way 2-5
- <30% cases and controls tested in the same way 1
- No cases and/or controls tested in the same way 0
- Not specified (any method or mixed percentage) 0

***Score for B2.3=B2.3 × 20/24 (maximum possible score is 20)***

***Overall score for B2 = (B2.1+B2.2+B2.3) x 25/42 (maximum possible score is 25)***

***Overall score for B = B1+B2 (maximum possible score is 40)***

**Section C: Confounding**

**C1. Adjustment for confounders in analysis Score from 0 to 22**

**(Select ONE or more options)**

- - No adjustment for confounders 0
  - Appropriate adjustment for confounders as **(Score additively) 0 to 22**

(*Note: Give full score if a confounder is controlled in analysis or*

*explicitly “tested for” a confounding effect i.e. if confounding has been*

*adjusted for via* ***matching*** *OR if a* ***multivariate*** *model has been used to*

*adjust for confounding in an unmatched study. If testing for all the*

*potential confounders” is reported but some are not mentioned by name,*

*give 50% of points for confounders whose names have not been mentioned.)*

1. Age 3
2. Gender 3
3. Smoking 3
4. Alcohol 3
5. Family history of oesophageal cancer 2
6. Pre-existing immunosuppression, prior to cancer diagnosis 2
7. Previous history of thoracic irradiation 2
8. Socio-economic status 2
9. Dietary intake high in processed/red meat, hot food/beverages, 1 to 2

pickled food; low in fresh fruits/vegetables
*(score 1 for any one or two, 2 for any three or four)*

**C2**. **Inappropriate adjustment for one or more confounders** **Score from** **-6 to 0**

**(Select ONE option)**

- - - That may create substantial bias -6
    - That may create some bias -3
    - No inappropriate adjustment for confounders 0

***Section C score = (C1 + C2) × 15 / 22* (**Maximum score is 15)***.***

**Section D: Analysis:**

**D1.** Appropriateness of main effect analysis **Score from** **0 to 3**

**(Select ONE option)**

- - - OR with confidence interval 3
    - OR without confidence interval 2
    - No OR calculated 0

***Score for section D = D1× 5/3* (**Maximum score is 5)***.***

***Total score = Section A + Section B + Section C + Section D***

References:

1. Barrie M, et al., Development of a scoring system to judge the scientific quality of information from case-control and cohort studies of nutrition and disease. *Nutr Cancer*, 1995;24(3):231-9.
2. Horwitz RI, Feinstein AR. Methodologic standards and contradictory results in Case-control research. *Am J Med*, 1979;66:556-64.
3. Realini JP, Goldzieher JW. Oral contraceptives and cardiovascular disease: a critique of the epidemiologic studies. *American Journal of Obstetrics and Gynecology*, 1985;152:729-19.
4. Chalmers TC, Smith H Jr, Blackburn B, et al. A method for assessing the quality of randomized control. *Controlled Clin Trial,* 1981;2:31-49.
